# Case Report: Successful Repair of Primary Aortoesophageal Fistula With an Endovascular Stent Graft and an Esophageal Stent

**DOI:** 10.3389/fsurg.2022.868663

**Published:** 2022-06-14

**Authors:** Zhi-Wei Wu, Yong-Dong Yao, Yi-Ming Li

**Affiliations:** ^1^ Department of Cardiology, The First Hospital of Putian, Putian, China; ^2^ Department of Emergency, The First Affiliated Hospital of Fujian Medical University, Fuzhou, China

**Keywords:** aortoesophageal fistula, endovascular stent, esophageal stent, case report, treatment

## Abstract

Aortoesophageal fistula (AEF), secondary to thoracic pseudoaneurysm as a result of upper gastrointestinal bleeding, is a rare condition and will be undoubtedly lethal without prompt surgical intervention. The estimated annual incidence of primary AEFs and secondary AEFs is about 0.0015% and 0.6%–2%, respectively. The challenges of the therapy posed by AEF are control of the hemorrhage, arterial reconstruction in an infection field, control of sepsis, and re-establishment of the alimentary tract. We present a case of a 58-year-old man who suffered from chest pain and hematemesis and was finally diagnosed with pAEF caused by descending thoracic pseudoaneurysm. Our team successfully deployed an endovascular stent graft and esophageal stent to seal ruptured thoracic aorta and esophageal defects, which provided a new surgical strategy for aortoesophageal fistula in the endovascular era.

## Introduction

Aortoesophageal fistula (AEF) was first described by Dubreuil (1) in 1818. The classical aortoesophageal syndromes of AEF, which were reported by Chiari (2), include a sequence of mid-thoracic pain or dysphagia, sentinel minor bleeding, and exsanguination after a symptom-free interval (3). An early careful evaluation and a high index of suspicion for the atypical hematemesis are crucial. In spite of various surgical interventions for AEF, few survivors have been reported. Therefore, there is no consensus or guidelines at present.

## Case Report

**Figure 1:**
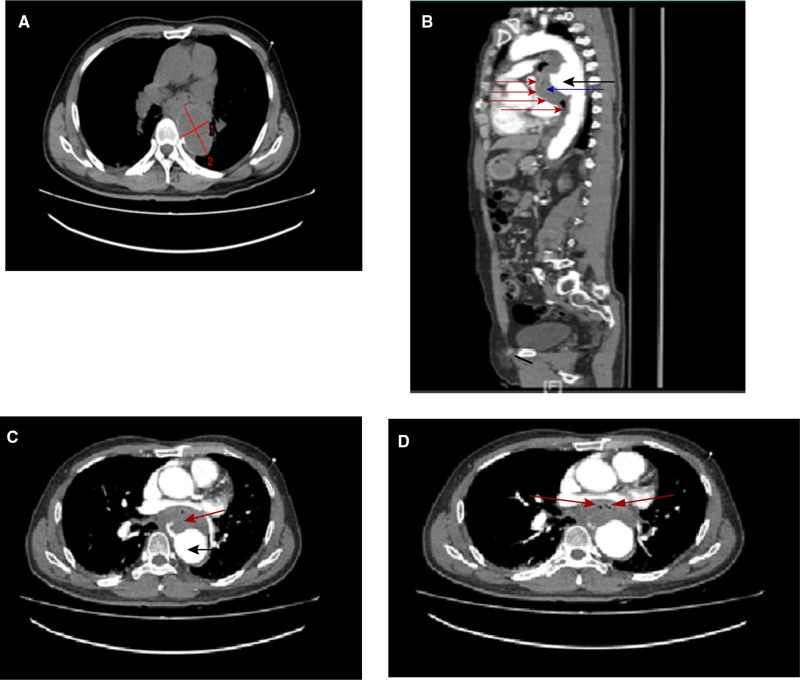
Computed tomography (CT) images showing (**A**) dilatation of descending aorta; the widest place reaches 4×6 cm (red line). Multidetector CT angiography: (**B**) sagittal reformatted images through the thorax showing active aortic bleeding generating an anterior hematoma (black arrow head) and exudate or rupture to form a large thrombus (blue arrow head), which is compressing the posterior wall of the esophagus (red arrow head), resulting in the abnormal course of the esophagus and is not recognizable in the picture. Multidetector CT angiography: (**C**) axial reformatted images through the thorax revealing active aortic bleeding generating an anterior hematoma (black arrow head) and exudate or rupture to form a large thrombus (red arrow head). (**D**) air bubbles (red arrow head) inside the aneurysm’s thrombus that are suggestive of esophageal erosion.

**Figure 2:**
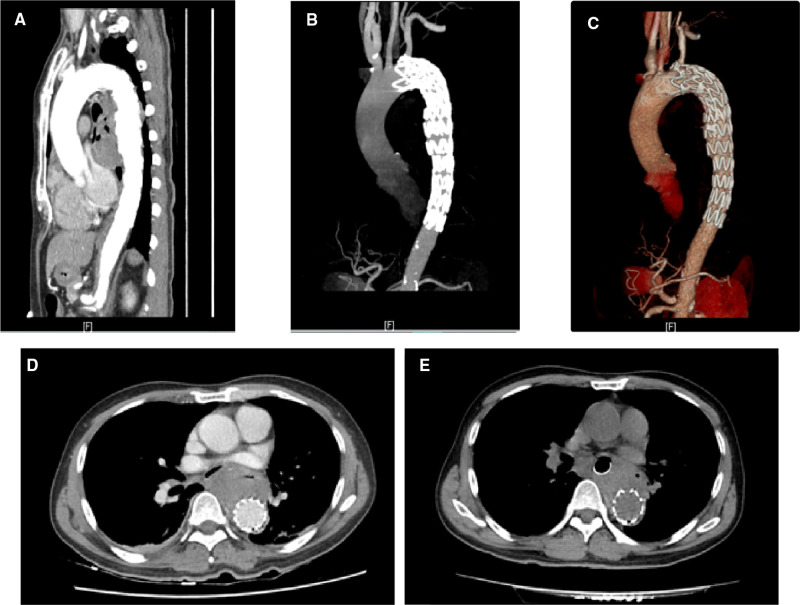
Postoperation computed tomographic angiography (**A**–**C**) and computed tomography (**D**) 1 day and (**E**) 14 days after AEF repair showing that the aortic lesions have been completely excluded, with no signs of endoleak, mediastinal infection, or stent-graft contamination.

**Figure 3:**
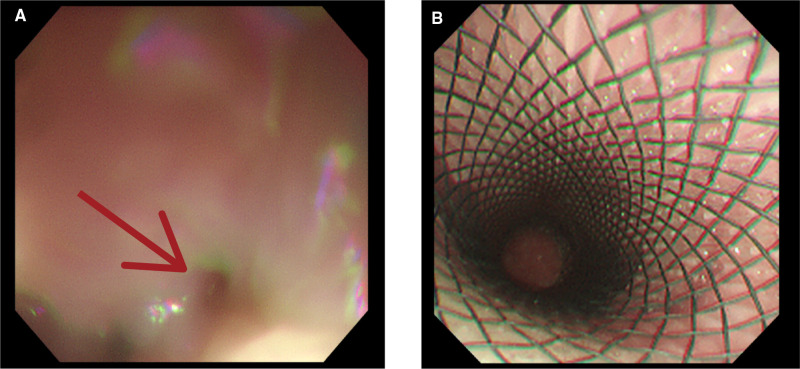
Upper gastrointestinal endoscopy revealing (**A**) an esophageal lesion (red arrow head) and (**B**) installation of an esophageal stent.

A 58-year-old male with a history of poorly controlled hypertension and hyperuricemia presented to a local hospital with complaints of chest pain for the past 1 month; then, aspirin was prescribed due to the change in the ST–T segment. The symptoms were still persistent, and he was admitted to the first-aid room of our institution with an episode of massive hematemesis from the last 12 h. He denied a history of abdominal pain, alcoholism, past history of gastrointestinal (GI) bleeding, or any significant past medical history. On physical examination, his heart rate (HR) was 86 beats/min with blood pressure (BP) of 138/81 mmHg, and the other laboratory indexes were unremarkable. Workup showed decreased hemoglobin (100 g/L) and hematocrit (0.295L/L), C-reactive protein (CRP) of 49 mg/L (normal range: <10.00 mg/L), and without coagulopathy. Surprisingly, plasma ammonia was normal. There was no history of melaena but persistent chest pain and normal plasma ammonia, which aroused our strong suspicion of an AEF. So, esophagigastroduodenoscopy (EGD) was canceled, and emergent computed tomography (CT) and angiography of the thorax (ACT) were performed. The aortogram revealed the presence of pseudoaneurysm (size 40 × 60 mm) in the descending thoracic aorta (Figure 1A). Urgently, the vascular surgical team was consulted, and thoracic endovascular aneurysm repair was done with a stent graft (Ankura) (Figure 2A–C); the outcome was favorable. Meanwhile, oral empirical treatment with moxifloxacin was initiated despite negative blood cultures. The patient was completely stable and underwent the treatment of esophageal lesion with the deployment of an esophageal stent on the fourth postoperative day (Figure 3). In addition, a gastrostomy tube was placed for postoperative transenteral nutrition. He received an oral intake of antibiotic therapy for 2 weeks until inflammation findings were negative. Postoperation computed tomographic investigation conducted on the first day and 14 days after AEF repairment suggested no esophageal leakage or grant infection (Figure 2D, E). He recovered well and was discharged 14 days after surgery. Two months after the discharge, the patient underwent the gastroscopy again, and the results showed that the fistula healed, the esophageal stent was removed, and the gastrostomy was closed. Gastroscope and chest CT were re-examined regularly every 3–6 months. There is no fever, chest pain, and other symptoms of infection. Now, the patient has recovered well and is back to normal life.

## Discussion

**Figure 4:**
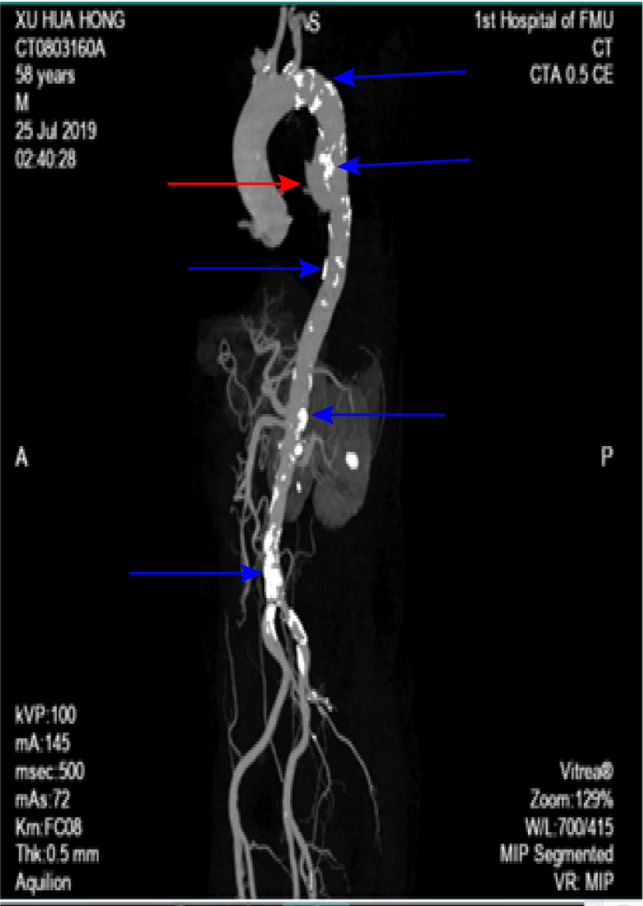
Three-dimensional reconstruction showing a descending thoracic aortic pseudoaneurysm (redarrow head) on the anterior aortic wall and multiple atheromatous plaques (blue arrow head).

AEF is a subtype of aortoenteric fistula since the lesion primarily involves the thorax rather than the abdomen, which constitutes approximately 10% of all aortoenteric fistulas (4). pAEF most frequently occurs between the duodenum and abdominal aorta. Sears and Scheltinga (5) received 81 published cases of aortoenteric fistulas and reported that it was most commonly located in the duodenum (54%), followed by the esophagus (28%). pAEF is rare and mostly secondary to a true aneurysm but less frequent to a pseudoaneurysm (6, 7). An additional rare cause of pAEF is penetrating aortic ulcer (PAU), which was first described in 1934 by Shennan (8, 9). To our best knowledge, this is the third case of AEF, which was caused by PAU. In our patient, there was no significant previous medical or surgical history, and the scan of computed tomography angiography showed a tortuous, atheromatous aorta with calcified plaques. Multiple parietal ulcers were observed on the descending aorta (Figure 4), which resulted from the ulceration of a previous atherosclerotic plaque. PAUs penetrate the aortic wall from the internal elastic lamina to the arterial media; the hematoma is initially contained by the tunica adventitia, leading to a pseudoaneurysm formation (10). Therefore, we can see a hematoma of 40 × 60 mm in the CTA due to active contrast media extravasation. According to Laplace’s equation, as the diameter of the aneurysm increases, the wall tension at any given intraluminal pressure increases and slowly weakens the aorta wall or ruptures the adventitia (11). These factors contribute to persistent extravasation of blood to form a large thrombus outside the pseudoaneurysm, which compresses the esophagus obviously (Figure 1B, C). In the thrombus, we also observed some free air bubbles, which suggested evidence of esophageal rupture (Figure 1D). The cause of esophageal rupture is that the esophageal wall is in contact with the high-pressure pulsating aorta for a long time, resulting in esophageal wall injury and ischemic necrosis of the esophageal wall. If AEF is suspected on medical history, symptoms, and work-up, the EGD must be avoided because of the high risk of fatal bleeding under the reduced intraesophageal pressure. On the contrary, angiography of the thorax should be performed immediately to confirm the diagnosis of AEF. Tokeno et al. (12) proposed the theory that AEF is essentially an infection in which the bacteria-free aorta becomes contaminated by material from the gastrointestinal tract. Urgent operation and strong, broad-spectrum antibiotic therapy offer the only chance for survival in these patients with AEFs. However, no consensus has been reached on therapeutic strategy because of the rarity of the disease and the changing therapeutic trends. Tokeno et al. (12) also reviewed articles published in the last decade concerning the trends in AEFs and showed that a treatment strategy combining graft replacement with esophagectomy has the most favorable prognosis among all the possible options.

A review of the literature in PubMed reveals nine published reports of nine primary AEFs caused by pseudoaneurysm or aneurysm who underwent the endovascular stent-graft repair (Table 1). Of these nine patients, one patient did not have any follow-up, four patients were doing well up to 36 months after the procedure (maximum follow-up time of 36 months, minimum follow-up time of 6 months), two patients died from an infection related to their stent graft (one from mediastinitis and one from sepsis), and two patients died from causes not related to infection of their stent graft (one patient from respiratory arrest and one from GI bleeding). The four patients who were reported to be alive did not suffer infections. Based on the published cases, first-stage graft replacement also faces a high risk of endograft infection, especially if there has been mediastinal contamination before operation (13). Thus, it has been suggested that thoracic endovascular aortic repair (TEAR) may be used as a damage control surgery for hemostasis as a bridge to graft replacement after an improvement in the patient’s general condition has been achieved (3). Moreover, Nishibe et al. (15) have reported a case in which the endurance of endovascular stents exceeded more than 3 years without any serious local or systemic complications. The strategy of repairment should be highly individualized and according to the capacity of the surgical team of the institution.

**Table 1:** Endovascular treatment of primary aortoesophageal fistulas.

Reference	Sex/Age	Etiology	Postop morbidities	Results/Followup
Leobon et al. (14)	M/80	Thoracic aortic aneurysm	Postoperative bleeding; re-intervention	Death from mediastinitis 25 M postop
Nishibe et al. (15)	F/71	Thoracic aortic aneurysm	None	Successful 36 M later
D’Ancona et al. (16)	F/78	Penetrating ulcer of mid-descending aorta	None	Successful 6 M later
Bonavina et al. (17)	M/79	Thoracic aortic aneurysm	Dyspnea, hemoptysis, bronchoesophageal fistula	Successful 9 M later
Zamora et al. (18)	F/71	Ingestion of foreign body (fish bone); thoracic aortic pseudoaneurysm	None	Successful 12 M later
Ting et al. (19)	M/87	Thoracic aortic pseudoaneurysm	Sepsis	Died from sepsis 3 M after procedure
Bos et al. (20)	Not mentioned	Thoracic aortic aneurysm	None	Death from unexplained respiratory arrest on POD2
Xia et al. (21)	M/66	Descending aortic pseudoaneurysm	Fever, bacterial infection	Died at 9 W from exsanguination
Zuber-Jerger et al. (22)	M/70	Thoracic aortic aneurysm	None	No follow-up reported

The patient, who was in the first-aid room at that moment, said only his wife was with him and his children were abroad. He experienced fear and anxiety from vomiting blood and chest pain, which would have been worse for him if he had undergone conventional general anesthesia. The patient was conscious during the procedure, and our staff were always comforting and encouraging, making him feel relaxed during the procedure.

In this case, our successful experience demonstrates the feasibility of the operation of pAEF using an endovascular stent graft and an esophageal stent. Compared to the traditional surgical treatment that includes resection or exclusion of aneurysms combined with external anatomical bypass surgery or *in situ* aneurysm repair, stent implantation is less invasive, especially in the acute phase of the disease. There are a variety of tubes for *in situ* replacement, including autologous veins, polytetrafluoroethylene grafts, cryopreserved allografts, and antibiotic-bound grafts. So, stent implantation showed its convenience. However, the mediastinum is a potential infection area and may cause a severe infection of vascular stents, which is the biggest limitation we are most concerned about. To our knowledge, there was no case reported to date about this scheme of operation. Large series cases and long-term follow-ups will be necessary to further establish the prospect of the proposed combined treatment of pAEF.

## Data Availability

The original contributions presented in the study are included in the article/supplementary material; further inquiries can be directed to the corresponding author/s.
